# Designing a Logistic Regression Model for a Dataset to Predict Diabetic Foot Ulcer in Diabetic Patients: High-Density Lipoprotein (HDL) Cholesterol Was the Negative Predictor

**DOI:** 10.1155/2021/5521493

**Published:** 2021-03-16

**Authors:** Seyyed Amir Yasin Ahmadi, Razieh Shirzadegan, Nazanin Mousavi, Ermia Farokhi, Maryam Soleimaninejad, Mehrzad Jafarzadeh

**Affiliations:** ^1^Student Research Committee, Iran University of Medical Sciences, Tehran, Iran; ^2^Social Determinants of Health Research Center, Lorestan University of Medical Sciences, Khorramabad, Iran; ^3^Student Research Committee, Lorestan University of Medical Sciences, Khorramabad, Iran; ^4^Faculty of Medicine, Babol University of Medical Sciences, Babol, Iran; ^5^Endocrine Research Center, Institute of Endocrinology and Metabolism, Iran University of Medical Sciences, Tehran, Iran

## Abstract

**Objectives:**

Although the risk factors for diabetic neuropathy and diabetic foot ulcer have been detected, there was no practical modeling for their prediction. We aimed to design a logistic regression model on an Iranian dataset to predict the probability of experiencing diabetic foot ulcers up to a considered age in diabetic patients.

**Methods:**

The present study was a statistical modeling on a previously published dataset. The covariates were sex, age, body mass index (BMI), fasting blood sugar (FBS), hemoglobin A1C (HbA1C), low-density lipoprotein (LDL), high-density lipoprotein (HDL), triglyceride (TG), insulin dependency, and statin use. The final model of logistic regression was designed through a manual stepwise method. To study the performance of the model, an area under receiver operating characteristic (AUC) curve was reported. A scoring system was defined according to the *beta* coefficients to be used in logistic function for calculation of the probability.

**Results:**

The pretest probability for the outcome was 30.83%. The final model consisted of age (*β*1 = 0.133), BMI (*β*2 = 0.194), FBS (*β*3 = 0.011), HDL (*β*4 = −0.118), and insulin dependency (*β*5 = 0.986) (*P* < 0.1). The performance of the model was definitely acceptable (AUC = 0.914).

**Conclusion:**

This model can be used clinically for consulting the patients. The only negative predictor of the risk is HDL cholesterol. Keeping the HDL level more than 50 (mg/dl) is strongly suggested. Logistic regression modeling is a simple and practical method to be used in the clinic.

## 1. Introduction

Diabetes mellitus (DM) is one of the major causes of morbidity and mortality in the world characterized by a rising blood glucose level. The prevalence of DM is estimated to be increased by 2050 [1]. This increasing prevalence is an alarm, because it imposes a high burden on the surveillance about DM complications. DM has many macrovascular and microvascular complications. A high glucose level results in both its conversion to sorbitol *via* the polyol pathway and formation of advanced glycosylated end products (AGE). The microvascular complications are attributed to many pathophysiological mechanisms that one of which is sorbitol accumulation in cells [2, 3]. Diabetic neuropathy is one of the microvascular complications of DM and is one of the most important causes of diabetic foot ulcers (other than the neuropathic ulcers, the ulcers can also be ischemic) [4].

A diabetic foot ulcer is a severe complication of DM that consists of damage to deep tissues of usually lower limbs along with neurological and peripheral vascular injures. Its incidence is globally increasing due to the increased prevalence of DM and increased life expectancy of DM patients. Therefore, it has a high global burden. Its lifetime prevalence is about 25% in DM patients, and it is estimated that one lower extremity is amputated due to DM every 30 seconds worldwide. In addition, its financial burden is of great importance. The average annual expenditure of diabetic foot ulcers is more than $8000 US per patient [5].

There are many risk factors and protecting factors for diabetic foot ulcers. A population-based cohort study showed that the risk factors were history of diabetic foot ulcer or amputation, insulin usage, gender, distal neuropathy, and foot deformity [6]. Researches were used to find the predictors of diabetic foot ulcers based on the risk and protecting factors. The attentions were to the commonly available clinical information [7].

Although the risk factors for diabetic neuropathy and diabetic foot ulcers have been detected, there was no simple practical modeling with a finally given formula for prediction. Hereby, we intend to perform a secondary analysis using binary logistic regression on an Iranian dataset to predict the probability of experiencing diabetic foot ulcers up to a specific age in diabetic patients.

## 2. Material and Methods

The present study was a statistical modeling on a previously published dataset in Lur and Lak populations of Iran. The dataset had been collected according to ethical guidelines, and it had an ethical registration number [8]. No further ethical registration was needed for this secondary analysis. The outcome variable was history of diabetic foot ulcers (as a binary variable). The covariates were sex, age, body mass index (BMI), fasting blood sugar (FBS), hemoglobin A1C (HbA1C), low-density lipoprotein (LDL), high-density lipoprotein (HDL), triglyceride (TG), insulin dependency, and statin use.

Stata 14 (StataCorp LLC, Texas, US) software was used for statistical modeling. To perform logistic regression, *-logit-* command was used and adjusted *beta* coefficients were reported. The model was repeated in 3 steps; step 1, all the covariates were imported to the model; step 2, the covariates with *P* value less than 0.1 in step 1 were imported to the model; and step 3, the covariates with *P* value less than 0.1 in step 2 were imported to the model. Then, the covariates of step 3 were considered as the final modeling. To study the performance of the model, the area under receiver operating characteristic (ROC) curve (AUC) was reported using *-lroc-* postestimation command. In addition, *-lsens-* postestimation command was used to report sensitivity and specificity at each cutoff point of the probability. A scoring system was defined using *-generate-* command according to the *beta* coefficients of step 3 of the model (equation (1)). Then, the scoring system was used in a univariable model to predict the outcome. This scoring system was imported to the sigmoid (logistic) function to predict the exact probability of the outcome variable (equation (2)). Marginal analysis on the sigmoid function was performed using *-margins*, *at* (score = (−8(1)5)) *plot-* postestimation command.

(1) $$\begin{array}{c} y=\beta 1.x1+\beta 2.x2+\beta 3.x3+\cdots +\beta 0=\text{score}, \end{array}$$

(2) $$\begin{array}{c} y=\frac{\exp\left(\text{score}\right)}{1+\exp\left(\text{score}\right)}=\text{probability}. \end{array}$$

## 3. Results

**Figure 1:**
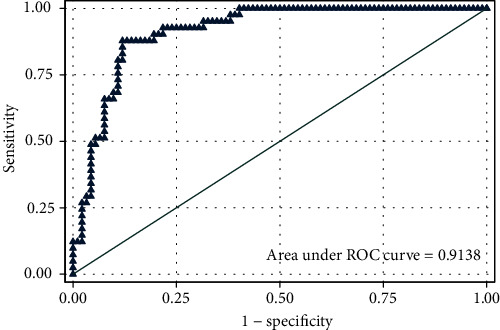
Postestimation ROC curve for the final step of the model (the unit of the predictor is the scoring system).

**Figure 2:**
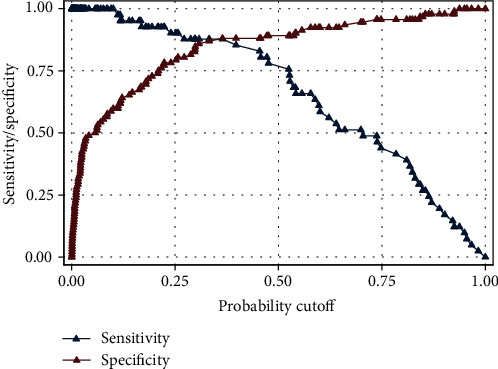
Postestimation sensitivity/specificity plot for the final step of the model (the unit of the predictor is the scoring system).

The pretest probability for the outcome was 30.83% in the samples of the present study. Logistic regression was performed in three steps. Among the covariates, sex, HbA1C, LDL, and TG could not pass step 1 (*P* > 0.1). Then, the covariate statin use could not pass step 2 (*P* > 0.1). The final model consisted of age (*β*1 = 0.133), BMI (*β*2 = 0.194), FBS (*β*3 = 0.011), HDL (*β*4 = −0.118), and insulin dependency (*β*5 = 0.986) (*P* < 0.1). The performance of the model was definitely acceptable (AUC = 0.914) with an acceptable preservation of sensitivity at higher cutoff points of the probability (Table 1, Figures 1 and 2).

**Figure 3:**
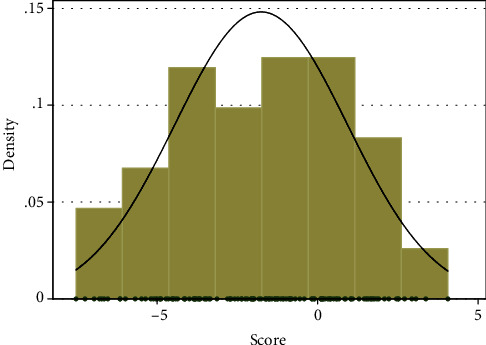
Distribution of diabetic foot ulcer predicting score in the studied cases. Mean = −1.759, standard deviation = 2.692, minimum = −7.525, maximum = 4.057.

**Figure 4:**
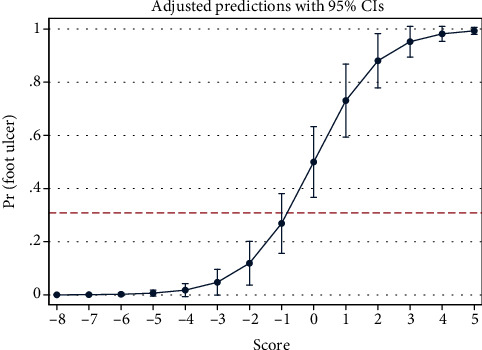
Marginal analysis shows the probability of diabetic foot ulcer at each score. The reference line shows the prevalence of diabetic foot ulcers in the studied samples 30.83% (pretest probability).

The final formula for the scoring system is shown (equation (3)). Distribution of the score for the samples of the study is also shown (Figure 3). The score of each diabetic patient should be replaced in logistic function (equation (2)) for prediction of experiencing diabetic foot ulcers up to a specific age. We imagined 10 examples for prediction of the probability (Table 2). The graph of this marginal prediction based on logistic function is shown (Figure 4). The number needed to treat (NNT) was calculated for improving HDL from 40 (mg/dl) to 50 (mg/dl) (equation (4)) for the 10 mentioned examples (Table 2). For instance, in a 70-year-old insulin-dependent patient with BMI = 30 (kg/m^2^) and FBS = 160 (mg/dl), at HDL = 40 (mg/dl), the probability of the outcome is 54.0% whereas at HDL = 50 (mg/dl), the probability of the outcome is 26.3% (NNT = 4):

(3) $$\begin{array}{c} y=0.1331305\times \text{age}+0.1944625\times \text{BMI}+0.0108864\times \text{FBS}+\left(-0.1184257\right)\times \text{HDL}+0.9855977\times \text{insulin }\left(0\text{ or }1\right)+\left(-12.98725\right), \end{array}$$

(4) $$\begin{array}{c} \text{NNT}=\frac{1}{\text{Absolute Risk Reduction}}=\frac{1}{P\left(\text{at HDL}1\right)-P\left(\text{at HDL}2\right)}. \end{array}$$

## 4. Discussion

This study was aimed at estimating the probability of experiencing diabetic foot ulcers at least for one time from the time of diagnosing DM up to the considered age. Accordingly, age, BMI, FBS, and insulin dependency were positive predictors while HDL was a negative predictor. This model seemed to be accurate enough, and since the *P* values of the final model were low, this model seemed to be repeatable for prospective use. Generally, in each regression model, reducing the number of covariates results in reduction of goodness of fit criteria such as *R* squared, pseudo-*R* squared, and AUC. Nevertheless, it is inevitable to remove covariates with high *P* values because of making nuisance in the model. In other words, although a chock-a-block with covariates model is more accurate for retrospective prediction of a currently studied sample, this model will not be repeatable for prospective prediction in another sample of population.

Before us, two other studies had used this dataset of ours for statistical modeling. Alfian et al. designed a deep neural network to predict diabetic retinopathy. Their model showed better performance than the previous modelings with accuracy of 82.03% [9]. Reddy et al. designed a neural network to predict diabetic neuropathy. Their aim was to compare different modeling methods [10].

Previously, there were not enough studies that modeled the predictors of diabetic foot ulcers. Boyko et al. tried to design a model using commonly available clinical information. They used Cox regression modeling. The advantages of their study in comparison to the study of ours were the cohort approach, higher sample size, and access to the time to events. Nevertheless, the disadvantages of their study in comparison to the study of ours were more complexity of the model, lack of studying lipid profile, lack of finding a protecting factor, and lack of reporting a final practical formula. The performance of our model was better according to the AUCs [7].

In many studies, low HDL and high TG were associated with increased diabetic peripheral neuropathy while LDL did not show any association. Smith et al. showed that there was an association between low HDL and elevated triglycerides with diabetic neuropathy. They aimed to determine whether the characteristics of metabolic syndrome other than hyperglycemia increased the risk of diabetic neuropathy [11]. Tesfaye et al., with the aim of investigating the risk factors for neuropathic modification, showed that the incidence of diabetic neuropathy was associated with high TG levels in addition to blood sugar [12]. A study conducted by Pai et al. was aimed at investigating the risk factors for peripheral neuropathy in patients with type 2 DM. They concluded that low levels of HDL increased the risk of diabetic peripheral neuropathy [13]. Rosales-Hernandez et al. after examining oxidized LDL (OxLDL) in diabetic peripheral neuropathy concluded that there was no association between its level and occurring peripheral neuropathy [14].

In contrast to the studies with positive results, Zhu et al. showed that the number of monocytes and HDL levels were similar between healthy individuals and patients with type 2 diabetes with or without diabetic peripheral neuropathy [15]. Interestingly, Li et al. in a study in China aimed at investigating the incidence of amputation in patients with diabetic foot ulcers and risk factors for amputation showed that low levels of TG were an independent risk factor for lower limb amputation in patients with diabetic foot ulcers [16].

Our study supported the results of previous studies for susceptibility to diabetic foot ulcers. Ikura et al. examined this issue of whether HDL levels predict the incidence of lower limb amputation and wound-related death in patients with diabetic foot ulcers or not. They concluded that low HDL levels in patients with diabetic foot ulcers were associated with the incidence of minor and major extremity amputation or wound-related death. But triglyceride and LDL levels did not predict them [17]. Pei et al. in a meta-analysis aimed to investigate the effect of lipids and lipoproteins on diabetic foot ulcer risk in patients with type 2 DM. They showed that decreased HDL was an associated factor [18]. Dai et al. conducted a study to investigate the relationship between vitamin D and risk of diabetic foot ulcer in patients with type 2 DM. The results of that study showed that low serum 25-OH-vitamin D levels were associated with the risk of diabetic foot ulcer. Although vitamin D levels showed higher diagnostic accuracy, the protective effect of HDL was greater based on logistic regression after adjusting the *beta* coefficients. The protective effects of HDL might be due to its anti-inflammatory effects on immune cells [19].

A high-fat diet results in hyperlipidemia. Cholesterol and other substances of lipid metabolism accumulate in neurons. The deposition of these substances causes oxidative stress, followed by increased expression of proinflammatory cytokines and neuronal apoptosis. An animal study showed that dyslipidemia was an independent risk factor for the development of diabetic neuropathy [20].

The strength of this study was achieving an acceptable performance (AUC > 0.90) and an acceptable goodness of fit (McFadden pseudo-*R*^2^ > 0.40) to be used in clinics. It seems that it was the first time that a practical formula for direct calculation of probability was reported for the prediction of diabetic foot ulcers. However, the study had some limitations. The most important one was lack of access to the time of event for this complication, and therefore, we did not consider time to event and could not perform Cox regression.

## 5. Conclusion

This model can be used clinically for consulting and managing diabetic patients who are at risk for diabetic foot ulcers. Among the predictors, age is not changeable and insulin dependency is usually inevitable; however, BMI and FBS can be controlled. The only negative predictor of the risk is HDL cholesterol. Keeping the HDL level more than 50 (mg/dl) is strongly suggested. Although statin use was not a significant predictor for diabetic foot ulcers, it should be regarded that its administration might be necessary for other indications and it can ameliorate lipid profile of the patients. Logistic regression modeling is a method in machine learning and data mining, but nevertheless, it is very practical and easy to interpret and use in daily clinic.

## Figures and Tables

**Table 1:** Logistic regression modeling for prediction of diabetic foot ulcer.

Predictor (unit)	*Beta* coefficient (*P* value)		
	Step 1	Step 2	Step 3
Sex (male)	0.314 (0.582)		
Age (year)	0.146 (<0.001^∗^)	0.136 (<0.001^∗^)	0.133 (<0.001^∗^)
BMI (kg/m^2^)	0.229 (0.003^∗^)	0.206 (0.002^∗^)	0.194 (0.004^∗^)
FBS (mg/dl)	0.014 (0.026^∗^)	0.010 (0.026^∗^)	0.011 (0.015^∗^)
HbA1C (%)	0.010 (0.641)		
LDL (mg/dl)	-0.174 (0.174)		
HDL (mg/dl)	-0.124 (0.012^∗^)	-0.124 (0.007^∗^)	-0.118 (0.010^∗^)
TG (mg/dl)	-0.323 (0.323)		
Insulin dependency (yes)	1.285 (0.038^∗^)	1.217 (0.039^∗^)	0.986 (0.074^∗^)
Statin use (yes)	-1.181 (0.092^∗^)	-0.929 (0.166)	
*Model properties and performance*			
Constant (*β*0)	-13.692	-12.497	-12.987
Number cases	133	133	133
Pseudo-*R* square	0.488	0.465	0.453
AIC	106.09	101.93	101.87
BIC	137.88	122.16	119.21
AUC	0.924	0.920	0.914

^∗^ *P* < 0.1. AIC: Akaike information criterion; BIC: Bayesian information criterion.

**Table 2:** Examples of diabetic foot ulcer prediction.

Example	Age	BMI	FBS	Insulin dependence	Probability (%) if			NNT (HDL 40➔50)
					HDL = 40	HDL = 45	HDL = 50	
1	50	25	140	0	1.0	0.5	0.2	157
2	50	25	140	1	2.4	1.3	0.8	60
3	70	25	140	0	11.7	6.8	3.9	13
4	70	25	140	1	26.2	16.4	9.8	6
5	50	30	140	0	2.4	1.3	0.7	61
6	50	30	140	1	6.2	3.5	2.0	24
7	70	30	140	0	26.0	16.3	9.7	6
8	70	30	140	1	48.5	34.2	22.3	4
9	70	30	160	0	30.3	19.4	11.8	5
10	70	30	160	1	54.0	39.2	26.3	4

Examples 1 and 2 indicate a middle-aged patient; examples 3 and 4 indicate an old-aged patient; examples 5 and 6 indicate a middle-aged patient with a high BMI; examples 7 and 8 indicate an old-aged patient with a high BMI; and examples 9 and 10 indicate an old-aged patient with a high BMI and a high FBS. The amounts of NNT have been rounded and calculated based on the exact amounts of probability.

## Data Availability

The raw data are available from https://data.mendeley.com/datasets/k62fdsnwkg/1.
